# Unraveling the Potential of Isorhamnetin as an Adjuvant in Depression Treatment with Escitalopram

**DOI:** 10.3390/cimb45090484

**Published:** 2023-09-21

**Authors:** Omar Gammoh, Esam Y. Qnais, Rabaa Y. Athamneh, Bilal Al-Jaidi, Deniz Al-Tawalbeh, Sara Altaber, Abdelrahim Alqudah, Alaa A. A. Aljabali, Murtaza M. Tambuwala

**Affiliations:** 1 Department of Clinical Pharmacy and Pharmacy Practice, Faculty of Pharmacy, Yarmouk University, Irbid 21163, Jordan; 2 Department of Biology and Biotechnology, Faculty of Science, The Hashemite University, Zarqa 13133, Jordan; esamqn@hu.edu.jo (E.Y.Q.); sara.taber12@gmail.com (S.A.); 3 Department of Medical Laboratory Sciences, Faculty of Allied Science, Zarqa University, Zarqa 13133, Jordan; rabaa_athamneh@yahoo.com; 4 Department of Medicinal Chemistry and Pharmacognosy, Faculty of Pharmacy, Yarmouk University, Irbid 21163, Jordan; bilal.aljaidi@yu.edu.jo (B.A.-J.); deniz.altawalbeh@yu.edu.jo (D.A.-T.); 5 Department of Clinical Pharmacy and Pharmacy Practice, Faculty of Pharmaceutical Sciences, The Hashemite University, Zarqa 13133, Jordan; abdelrahim@hu.edu.jo; 6 Department of Pharmaceutics and Pharmaceutical Technology, Faculty of Pharmacy, Yarmouk University, Irbid 21163, Jordan; alaaj@yu.edu.jo; 7 Lincoln Medical School, University of Lincoln, Brayford Pool Campus, Lincoln LN6 7TS, UK

**Keywords:** isorhamnetin, escitalopram, antidepressant, lipopolysaccharide, Nrf2, BDNF, HO-1, oxidative stress, inflammation

## Abstract

Oxidative stress and inflammation are implicated in depression. While selective serotonin reuptake inhibitors (SSRIs) like escitalopram are commonly prescribed as first-line treatments, their inconsistent efficacy and delayed onset of action necessitates the exploration of adjunctive therapies. Isorhamnetin, a flavonol, has shown antioxidant and anti-inflammatory properties that makes exploring its antidepressant effect attractive. This study aims to investigate the adjuvant potential of isorhamnetin in combination with escitalopram to enhance its antidepressant efficacy in a lipopolysaccharide (LPS)-induced depression model using Swiss albino mice. Behavioral paradigms, such as the forced swim test and open field test, were employed to assess depressive symptoms, locomotion, and sedation. Additionally, enzyme-linked immunosorbent assays were utilized to measure Nrf2, BDNF, HO-1, NO, and IL-6 levels in the prefrontal cortex and hippocampus. The results demonstrate that isorhamnetin significantly improves the antidepressant response of escitalopram, as evidenced by reduced floating time in the forced swim test. Moreover, isorhamnetin enhanced antidepressant effects of escitalopram and effectively restored depleted levels of Nrf2, BDNF, and HO-1 in the cortex caused by LPS-induced depression. Isorhamnetin shows promise in enhancing the efficacy of conventional antidepressant therapy through antioxidant and anti-inflammatory effects.

## 1. Introduction

Depression is a disorder prevalent worldwide and is associated with significant disabilities [1]. The pathology of depression involves complex interactions between various factors, including deficiency of monoamines, oxidative stress, and inflammation [2]. Several antidepressant classes exist such as selective serotonin reuptake inhibitors (SSRIs), serotonin norepinephrine reuptake inhibitors (SNRIs), tricyclic antidepressants (TCAs), and others. These medications act by enhancing the concentrations of the relevant monoamines (serotonin, dopamine, and norepinephrine) at the synaptic cleft. For example, escitalopram, a famous SSRI, is considered an effective maintenance treatment for depression; however, its latency and inconsistent efficacy have highlighted the need for augmentation with molecules that exhibit different mechanisms of action, such as anti-inflammatory and antioxidant compounds [3]. In this context, the cross-talk between depression and oxidative stress/inflammation, particularly the role of nuclear factor erythroid 2-related factor 2 (Nrf2) and its target genes, has emerged as an attractive area of investigation Numerous studies have established the association between increased interleukin-6 (IL-6) levels and depression [4]. IL-6 activation has been linked to the induction of various inflammatory mediators, including nitric oxide (NO), which is well established in depression. Patients with depression show elevated NO levels compared to controls, and animal models of depression exhibit increased NO levels that normalize with antidepressant treatment [5,6,7]. These findings suggest a potential role for NO in modulating the inflammatory and redox state in depression. Nrf2, a key regulator of these processes, has been shown to modulate various cytoprotective and antioxidant intracellular players, including Brain-Derived Neurotrophic Factor (BDNF) and Heme Oxygenase-1 (HO-1) [8,9]. Activation of Nrf2 by certain compounds, such as curcumin, has demonstrated antidepressant effects in mice models, while downregulation of Nrf2 has been observed after lipopolysaccharide (LPS) challenge [10,11]. Despite the evidence supporting the involvement of Nrf2 in the antidepressant effects of certain compounds, little research has explored the antidepressant potential of isorhamnetin (ISO), a flavonol aglycone abundant in several medicinal plants. Several medicinal plants have shown promising antidepressant and anxiolytic activities in both preclinical and clinical trials. Those plants have also been used in traditional medicine [12,13]. The most abundant compounds in aforementioned plants are the flavonols and flavonoid glycosides such as quercetin, isorhamnetin, and kaempferol [14].

Isorhamnetin (ISO) has shown anti-inflammatory, antitumor, antioxidant, brain-protective, and memory-enhancing properties in mice [15] and antitumor and anti-oxidant properties [16], brain protection against ischemic injury [17], and memory-enhancing capacity in mice [18]; however, the study of its mood-enhancing properties is still in its emerging stage [19]. Evidence also suggests that ISO may activate Nrf2 and its target proteins, including BDNF and HO-1, while modulating NO and IL-6 levels [20,21,22]. Moreover, ISO was found to modulate NO and IL-6 levels [23,24]. This study aims to investigate whether isorhamnetin can act as an adjuvant antidepressant with escitalopram in an LPS-induced model of depression. This study explored the changes in the expressions of Nrf2, BDNF, HO-1, NO, and IL-6 in the prefrontal cortex and the hippocampus.

This study employed a randomized, controlled animal model using LPS-induced depression. Male mice were divided into five groups: control, LPS-induced depression, LPS + ISO, LPS+ escitalopram, and LPS + ISO + escitalopram. Behavioral assessments were conducted using established depression and anxiety tests, and oxidative stress/inflammatory markers were measured in the hippocampus and prefrontal cortex. Additionally, the expressions of Nrf2, BDNF, HO-1, NO, and IL-6 were analyzed using enzyme-linked immunosorbent assays. Based on the literature review, it was hypothesized that the combination of ISO with escitalopram might result in a greater reduction of depressive behaviors compared to either treatment alone. Moreover, ISO was expected to modulate Nrf2-regulated cytoprotective and antioxidant genes and, subsequently, their corresponding proteins, leading to improvements in the redox state and reduction in oxidative stress markers. The study aimed to provide novel insights into the antidepressant potential of ISO and its underlying molecular mechanisms.

## 2. Materials and Methods

### 2.1. Animals

Male Swiss albino mice from the Animal House Facility of The Hashemite University, Zarqa, Jordan, aged 6–8 weeks and weighing 25 g, were selected as the experimental subjects for the present study. The mice were housed individually in separate cages under controlled environmental conditions, maintaining a temperature of 25 °C with 50–60% humidity and continuous air ventilation. Mice were exposed to 12 h light/12 h dark cycle, and all behavioral tests were carried out between 9:00 and 14:00. Ethical guidelines for the care and use of laboratory animals were strictly adhered to during the study, with approval from the Yarmouk University Institutional Review Board (IRB) committee and the Dean of Scientific Research (Project Number: 51/2022).

### 2.2. Study Design and Treatments

A preliminary pilot investigation was conducted to explore the antidepressant properties of isorhamnetin using the forced swim test, to choose the optimal LPS dose for the recruited mice and to validate the experimental settings and efficacy of escitalopram as the positive control. Mice were intraperitoneally injected with isorhamnetin (50 mg/kg), and two hours later, they underwent the forced swim test. The results were compared to those of the positive control, escitalopram. Subsequently, for the main experiment, an acute LPS-induced model was employed to evaluate the effects of both escitalopram and isorhamnetin. Initially, the treatments were administered at 9 am, and two hours later, LPS was injected, and two hours after that behavioral paradigms were performed. The following groups were studied: control, LPS, LPS + Iso, LPS + Esc, and LPS + Esc + Iso. Isorhamnetin (50 mg/kg) and escitalopram (10 mg/kg) were intraperitoneally administered using a procedure provided in [25,26] with modifications, followed by a single dose of LPS injection (2 mg/kg) as mentioned in [27]. Behavioral paradigms were performed two hours after LPS injection, just before scarification. LPS was freshly prepared on the same day of administration using saline. Each group consisted of 4–6 animals. Escitalopram, in pure powder form, was supplied as a general gift from Pharma International Company. Isorhametin and LPS were purchased from Santa Cruz.

### 2.3. Behavioral Paradigms

#### 2.3.1. Forced Swim Test

The forced swim test (FST) was utilized to assess depressive behaviors in the mice, following a widely accepted procedure [28]. Briefly, individual mice were placed in an open glass chamber filled with water to a height of 15 cm, and maintained at a constant temperature of 26 ± 1 °C. The test duration was 5 min, during which the floating time (FT), defined as the duration of complete immobility in the water, was measured. A higher FT value is indicative of greater depressive behavior.

#### 2.3.2. Open Field Test

To assess locomotion activity, sedation, and anxiety in the mice, the open field test (OFT) was performed [29]. The mice were placed in a central square of the open field maze and allowed to move freely for 5 min. The test room was appropriately illuminated, and noise and distractions were minimized to ensure a controlled environment. The number of lines crossed and rearing frequency represented anxiety and locomotion activity.

### 2.4. Biochemical Assays

ELISA kits (Sunlon Biotech Co Ltd., Hangzhou, China) were utilized to quantify the expression levels of Nrf2, BDNF, HO-1, and IL-6 in the hippocampus and cortex, following the manufacturer’s instructions. Additionally, NO levels were determined using a commercially available colorimetric kit (Sunlon Biotech Co Ltd., Hangzhou, China) based on the Griess reaction.

### 2.5. Data Analysis

Statistical analysis was conducted using a one-way ANOVA followed by Tukey’s post hoc test to compare the data obtained from the behavioral tests and biochemical assays. The significance level was set at *p* < 0.05. The results are presented as the mean ± standard error of the mean (SEM).

## 3. Results

### 3.1. Forced Swim Test

**Figure 1:**
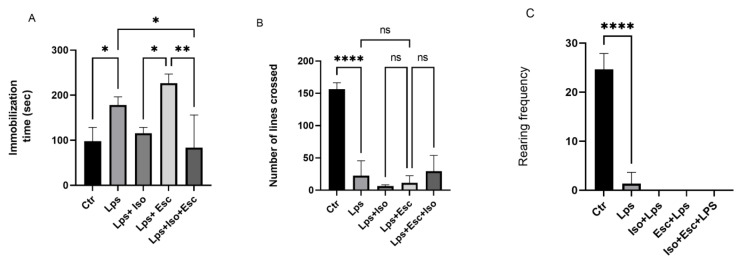
(**A**) Forced swim test. Floating time among the different groups. ANOVA followed by Tukey’s post hoc analysis. Values are expressed as the mean ± SEM (ANOVA followed by Tukey’s test). F(4, 14) = 10.43; *p* < 0.001,* *p* < 0.05, ** *p* < 0.001. Ctr: control; Lps: lipopolysaccharide, Iso: isorhamnetin, Esc: escitalopram; ns: non-significant; and SEM, standard error of the mean. (**B**). Open field test number of lines crossed among the different groups. ANOVA followed by Tukey’s post hoc analysis. Values are expressed as the mean ± SEM (ANOVA followed by Tukey’s test). F(4, 10) = 43.27; *p* < 0.0001, **** *p* < 0.0001. Ctr: control; Lps: lipopolysaccharide Iso: isorhamnetin, Esc: escitalopram; and SEM, standard error of the mean. (**C**). Open field test rearing frequency among the different groups. ANOVA followed by Tukey’s post hoc analysis. Values are expressed as the mean ± SEM (ANOVA followed by Tukey’s test). F (4, 10)= 113.7; *p* < 0.0001, **** *p* < 0.0001. Ctr: control; Lps: lipopolysaccharide, Iso: isorhamnetin, Esc: escitalopram; ns: non-significant; and SEM, standard error of the mean.

The results of the Forced Swim Test (FST) were used to evaluate the antidepressant efficacy of the treatments. Mice treated with LPS demonstrated a significant increase (*p* < 0.05) in floating time, indicative of significant depression. However, mice treated with LPS + Iso did not show any significant variation from the control group. Mice treated with LPS + Esc exhibited a significant (*p* < 0.05) deterioration in floating time compared to the control group. Notably, mice treated with LPS + Esc + Iso demonstrated a significant (*p* < 0.05) improvement in floating time compared to the LPS-treated group and exhibited floating times similar to the control group (Figure 1).

### 3.2. Open Field Test

The number of lines crossed, reflecting the anxiety, locomotor activity, and sedation of the animals, showed a significant (*p* < 0.05) reduction in the LPS-treated mice compared to the control. Also, all the other groups did not show any activity. Furthermore, the rearing frequency (RF), reflecting the severity of sedation, demonstrated a significantly diminished (*p* < 0.05) trend in the LPS-treated group versus control, and all the treatment groups that received LPS (Figure 1).

### 3.3. Nrf2 Expression

**Figure 2:**
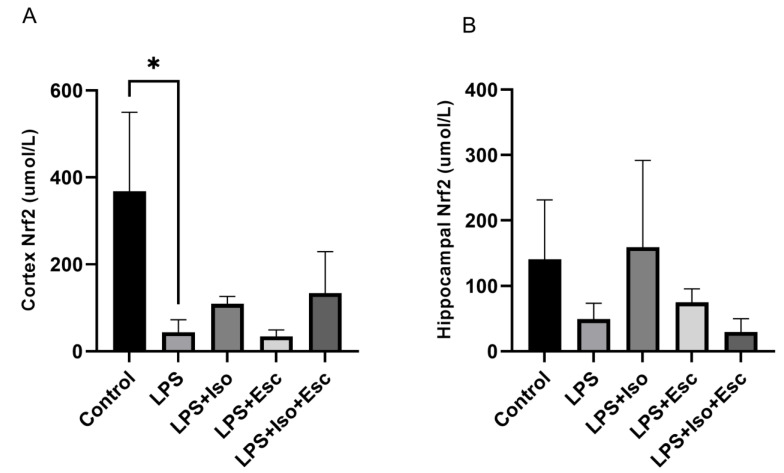
(**A**) Nrf2 expression in the cortex among the different groups. ANOVA followed by Tukey’s post hoc analysis. Values are expressed as the mean ± SEM (ANOVA followed by Tukey’s test). F(4, 11) = 6.23; *p* = 0.007. * *p* < 0.05. Ctr: control; Lps: lipopolysaccharide, Iso: isorhamnetin, Esc: escitalopram; and SEM, standard error of the mean. (**B**) Hippocampal Nrf2 expression among the different groups. ANOVA followed by Tukey’s post hoc analysis. Values are expressed as the mean ± SEM (ANOVA followed by Tukey’s test). F(4, 11) = 6.23; *p* = 0.007. * Ctr: control; lps: Lipopolysaccharide, Iso: isorhamnetin, Esc: escitalopram; and SEM: standard error of the mean.

The expression of Nrf2 was quantified in the cortex and hippocampus using ELISA. In the cortex, LPS-treated group exhibited significantly lower (*p* < 0.05) Nrf2 expression compared to the control group. In contrast, mice receiving the combination treatment of LPS + Esc + Iso did not show any decline (*p* > 0.05) in the expression of Nrf2. However, in the hippocampus, no significant changes (*p* > 0.05) in Nrf2 expression were observed among the study groups, although a decreasing trend was observed in the LPS + Esc + Iso group (Figure 2).

### 3.4. BDNF Expression

**Figure 3:**
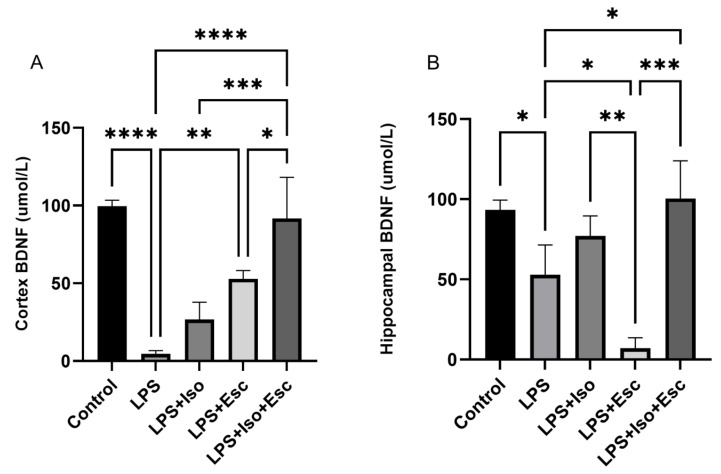
(**A**) BDNF expression in the cortex among the different groups. ANOVA followed by Tukey’s post hoc analysis. Values are expressed as the mean ± SEM (ANOVA followed by Tukey’s test). F(4, 10) = 28.69; *p* < 0.0001. * *p* < 0.05, ** *p* < 0.01, *** *p* < 0.0001, **** *p* < 0.0001. Ctr: control; Lps: lipopolysaccharide, Iso: isorhamnetin, Esc: escitalopram; ns: non-significant; and SEM: standard error of the mean. (**B**) Hippocampal BDNF expression among the different groups. ANOVA followed by Tukey’s post hoc analysis. Values are expressed as the mean ± SEM (ANOVA followed by Tukey’s test). F(4, 10) = 18.70; *p* < 0.0001, * *p* < 0.05, ** *p* < 0.01, *** *p* < 0.0001. Ctr: control; Lps: lipopolysaccharide, Iso: isorhamnetin, Esc: escitalopram; and SEM: standard error of the mean.

The expression of BDNF was measured in the cortex and hippocampus using ELISA. In the cortex, BDNF was significantly (*p* < 0.0001) diminished in the LPS-treated group compared to the control. All other groups showed similar expressions (*p* > 0.05). In the hippocampus, BDNF expression was significantly (*p* < 0.05) lower in the LPS-treated group compared to the control; however, it was normalized with the LPS + Esc + Iso group (Figure 3).

### 3.5. HO-1 Expression

**Figure 4:**
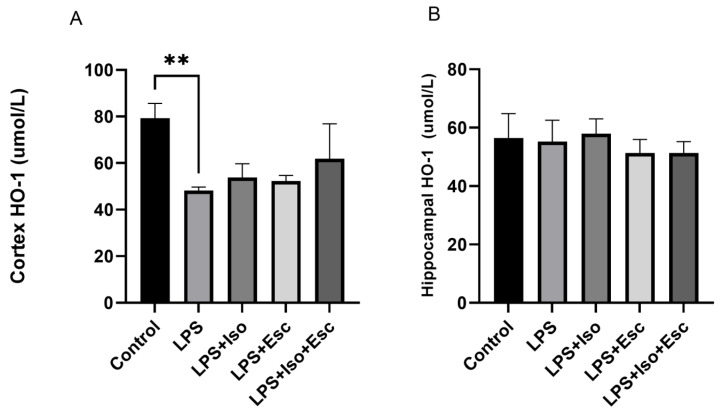
(**A**) HO-1 expression in the cortex among the different groups. ANOVA followed by Tukey’s post hoc analysis. Values are expressed as the mean ± SEM (ANOVA followed by Tukey’s test). F(4, 10) = 7.39; *p* = 0.005, ** *p* < 0.01. Ctr: control; Lps: lipopolysaccharide, Iso: isorhamnetin, Esc: escitalopram; and SEM: standard error of the mean. (**B**) Hippocampal HO-1 expression among the different groups. ANOVA followed by Tukey’s post hoc analysis. Values are expressed as the mean ± SEM (ANOVA followed by Tukey’s test). F(4, 10) = 0.70; *p* = 0.58. Ctr: control; Lps: lipopolysaccharide, Iso: isorhamnetin, Esc: escitalopram; and SEM: standard error of the mean.

The expression of HO-1 was measured in the cortex and hippocampus using ELISA. In the cortex, HO-1 expression was significantly (*p* < 0.05) decreased with LPS treatment, and it was only normalized in the LPS + Iso + Esc treatment group, showing no significant difference when compared with the control group (*p* > 0.05). However, in the hippocampus, HO-1 expression did not vary significantly among the study groups (Figure 4).

### 3.6. NO Levels

**Figure 5:**
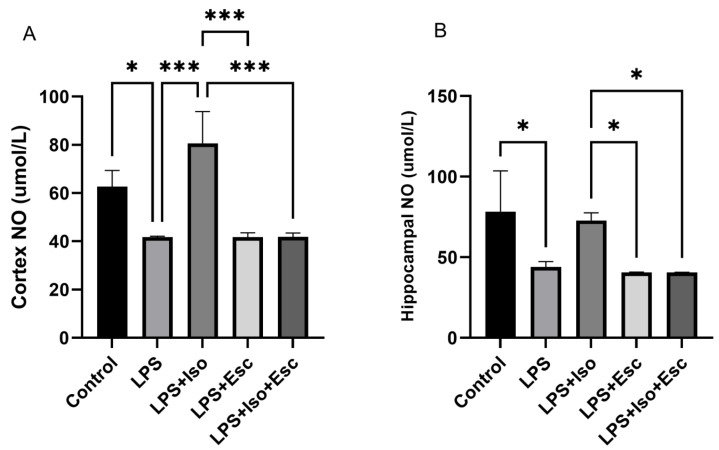
(**A**) NO expression in the cortex among the different groups. ANOVA followed by Tukey’s post hoc analysis. Values are expressed as the mean ± SEM (ANOVA followed by Tukey’s test). F(4, 10) = 20.61; *p* < 0.0001. * *p* < 0.05, *** *p* < 0.001. Ctr: control; Lps: lipopolysaccharide, Iso: isorhamnetin, Esc: escitalopram; and SEM: standard error of the mean. (**B**) Hippocampal NO expression among the different groups. ANOVA followed by Tukey’s post hoc analysis. Values are expressed as the mean ± SEM (ANOVA followed by Tukey’s test). F(4, 10) = 7.78; *p* = 0.004. * *p* < 0.05. Ctr: control; Lps: lipopolysaccharide, Iso: isorhamnetin, Esc: escitalopram; and SEM: standard error of the mean.

The measurement of NO levels in the cortex revealed that they were significantly decreased (*p* < 0.05) in the LPS group compared to the control group. Similarly, in the hippocampus, NO levels were significantly decreased (*p* < 0.05) in the LPS group compared to the control group (Figure 5).

### 3.7. IL-6 Expression

**Figure 6:**
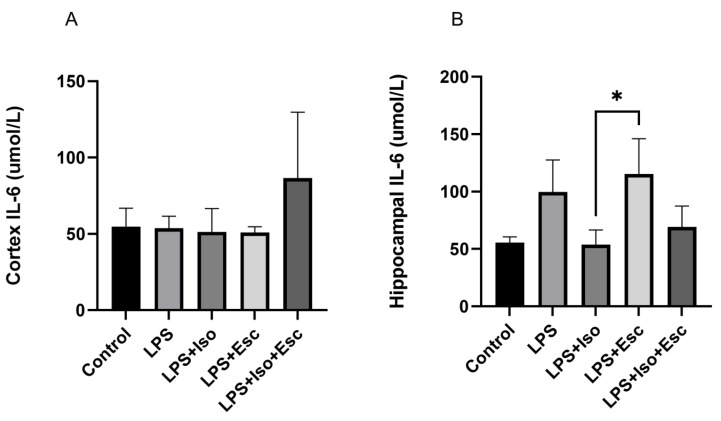
(**A**) IL-6 expression in the cortex among the different groups. ANOVA followed by Tukey’s post hoc analysis. Values are expressed as the mean ± SEM (ANOVA followed by Tukey’s test). F(4, 13) = 1.82; *p* = 0.18, Ctr. Ctr: control; Lps: lipopolysaccharide, Iso: isorhamnetin, Esc: escitalopram;;and SEM: standard error of the mean. (**B**) Hippocampal IL-6 expression among the different groups. ANOVA followed by Tukey’s post hoc analysis. Values are expressed as the mean ± SEM (ANOVA followed by Tukey’s test). F(4, 12) = 6.53; *p* = 0.005, * *p* < 0.05. Ctr: control; Lps: lipopolysaccharide, Iso: isorhamnetin, Esc: escitalopram; and SEM: standard error of the mean.

The expression of IL-6 was measured in the cortex and hippocampus using ELISA. In the cortex, IL-6 expression did not vary significantly (*p* > 0.05) among the study groups. However, in the hippocampus, IL-6 was significantly (*p* < 0.05) increased in the LPS + Esc treated group compared to the control (Figure 6).

## 4. Discussion

The current study aimed to explore the potential adjuvant antidepressant effect of isorhamnetin with escitalopram in an LPS model, while also investigating changes in Nrf2, BDNF, HO-1, NO, and IL-6 in the prefrontal cortex and the hippocampus. We report that isorhamnetin can augment the efficacy of escitalopram during early inflammatory depressive state. This finding opens new horizons to further study bridging the late onset of action of antidepressants.

Depression is a complex mental disorder affecting millions of individuals worldwide, characterized by persistent sadness, cognitive impairment, and loss of interest in activities. While selective serotonin reuptake inhibitors (SSRIs), such as escitalopram, are commonly used as first-line antidepressants, their limited efficacy and delayed onset of action highlight the need for potentiation using molecules acting through different mechanisms, including antioxidants and anti-inflammatory agents. Preclinical studies have demonstrated the antidepressant potential of certain flavonoids, including isorhamnetin, in animal models of depression. A recent study highlighted the antidepressant effects of isorhamnetin in an LPS-induced depression and anxiety model [19]. In this context, our study sought to explore the role of isorhamnetin as an adjuvant to escitalopram in the treatment of depression.

The present study aimed to explore the potential adjuvant antidepressant effect of isorhamnetin in combination with escitalopram using an LPS model. Additionally, the study sought to investigate changes in Nrf2, BDNF, HO-1, NO, and IL-6 in the prefrontal cortex and hippocampus.

Our results revealed that the addition of isorhamnetin significantly improved the antidepressant efficacy of escitalopram. Notably, there was a different expression in the levels of Nrf2, BDNF, and HO-1 in the cortex compared to the hippocampus.

Considering that escitalopram, like other SSRIs, may have limitations such as inconsistent efficacy and delayed onset of action, there is a growing need to explore potentiation using molecules that act via different mechanisms, including antioxidants and anti-inflammatory compounds. While the antidepressant role of some flavonoids has been previously studied in animal models, the investigation of isorhamnetin’s antidepressant properties was highlighted in only one recent study that emerged during the preparation of this manuscript [16]. In this study, isorhamnetin demonstrated positive effects in alleviating depression and anxiety in an LPS model [19].

Our findings suggest that the beneficial effects of isorhamnetin when combined with escitalopram may be attributed to its antioxidant and anti-inflammatory properties. Previous research has shown that isorhamnetin can suppress LPS-induced oxidative stress in vitro [30,31], and decrease oxidative stress levels in the brain of mice. Furthermore, isorhamnetin and similar flavonols have been found to normalize proinflammatory cytokines such as TNF-α and IL-1β in both the periphery and the brain of experimental animals or plasma samples from patients diagnosed with depression [32].

Moreover, our results demonstrated that the downregulation of Nrf2 expression in the cortex induced by LPS was consistent with existing literature, as LPS exhibits a potent inflammatory action that overwhelms the cellular antioxidant defense mechanisms [19,33]. Nrf2, as a master regulator for many downstream cytoprotective and antioxidant cellular players, including BDNF and HO-1, appeared to be influenced by an Nrf2-dependent mechanism behind the downregulation of BDNF and HO-1.

Furthermore, our findings support the notion that the combination of isorhamnetin and escitalopram activates Nrf2, HO-1, and BDNF expression in the cortex, thereby inhibiting LPS-induced inflammation. This observation aligns with previous studies.

It is well known that LPS, a bacterial toxin, exhibits a potent inflammatory action that overwhelms the cellular antioxidant defensive mechanisms, this explains the sharp decline in Nrf2 expression in our study, our finding is consistent with previous studies [34]. Also, Nrf2 is a master regulator for many downstream cytoprotective and antioxidant cellular players such as BDNF and HO-1 [8,9,34,35]. In the present study, we suggest an Nrf2-dependent mechanism behind the downregulation of BDNF and HO-1 [8,9,35].

In contrast, the results from the hippocampus displayed a different pattern of Nrf2, BDNF, and HO-1. Nrf2 expression did not exhibit significant changes across the study groups. Although BDNF was downregulated in the LPS group, it was upregulated and restored to normal levels with the combined treatment of isorhamnetin and escitalopram. This differential response in the hippocampus may be attributed to the acute inflammation design employed in our study, possibly limiting the timeframe for treatments to exert changes in hippocampal expressions compared to the frontal cortex [30,36].

Additionally, our results indicated that NO levels were decreased in both the cortex and hippocampus under the different treatments, but they were not upregulated with any of the interventions. This finding is consistent with the existing literature, suggesting that isorhamnetin exhibits antioxidant effects by inhibiting the expression of inducible nitric oxide synthase (iNOS), a key regulator of NO synthesis during LPS-induced inflammation.

Our results also demonstrated that NO levels were decreased in the cortex and hippocampus without being upregulated under the different treatments. This is consistent with existing literature indicating that ISO exhibits antioxidant effects by inhibiting the expression of the inducible nitric oxide synthase (iNOS), the key regulator of NO synthesis during LPS-induced inflammation [23,37,38].

The presented study investigated the possibility of acute changes in IL-6 levels in the cortex and the hippocampus under acute LPS insult. The cortical levels of IL-6 did not vary across the study groups; however, in the hippocampus, the LPS + Esc treated group demonstrated a significant elevation in IL-6 that was normalized in the Esc + ISO group. The increase in IL-6 in depressed patients has been already established [39], indicating a strong association of this pro-inflammatory marker with depression. Our finding is consistent with previous literature as our behavioral results showed that Esc was unable to completely reverse the depressive symptoms induced by LPS, and on the other hand, the addition of ISO resulted in improvement of depression and normalization of IL-6 level similar to the control group.

Although our study makes a valuable contribution to the literature by identifying isorhamnetin as a potential antidepressant adjuvant to escitalopram, particularly in restoring Nrf2, BDNF, and HO-1 in the frontal cortex, we acknowledge certain limitations. For instance, the use of a single high dose of LPS and the assessment of behavior within a short timeframe reflect an early inflammation-induced depression model [19], similar to previous studies. However, future research may benefit from exploring other chronic stress models to evaluate the antidepressant effects of isorhamnetin. Furthermore, our study focused on isorhamnetin in combination with escitalopram, and future investigations could extend to explore isorhamnetin with other classes of antidepressants to better understand potential synergistic effects.

## 5. Conclusions

In conclusion, our study demonstrates that isorhamnetin significantly enhances the antidepressant efficacy of escitalopram. Moreover, isorhamnetin restores the diminished expressions of Nrf2, BDNF, and HO-1 in the cortex. These findings suggest a potential role for isorhamnetin as an adjuvant in depression treatment, warranting further investigation in both preclinical and clinical settings. Future studies could delve deeper into the specific mechanisms of action and explore the translational potential of this novel combination therapy for depression management.

## Data Availability

Data are available from the corresponding author upon request.
